# Multimodal Data and Machine Learning for Detecting Specific Biomarkers in Pediatric Epilepsy Patients With Generalized Tonic-Clonic Seizures

**DOI:** 10.3389/fneur.2018.01038

**Published:** 2018-12-10

**Authors:** Jianping Wang, Yongxin Li, Ya Wang, Wenhua Huang

**Affiliations:** ^1^The Second Affiliated Hospital of Guangzhou Medical University, Guangzhou, China; ^2^Guangdong Provincial Key Laboratory of Medical Biomechanics, School of Basic Medical Sciences, Southern Medical University, Guangzhou, China

**Keywords:** generalized tonic-clonic seizures, epilepsy children, voxel-based morphometry, gray matter volume, resting-state fMRI, fractional ALFF, support vector machine

## Abstract

Previous neuroimaging studies of epilepsy with generalized tonic-clonic seizures (GTCS) focus mainly on adults. However, the neural mechanisms that underline this type of epilepsy remain unclear, especially for children. The aim of the present study was to detect the effect of epilepsy on brains of children with GTCS and to investigate whether the changes in the brain can be used to discriminate between epileptic children and healthy children at the level of the individual. To achieve this purpose, we measured gray matter (GM) volume and fractional amplitude of low-frequency fluctuation (fALFF) differences on multimodel magnetic resonance imaging in 14 children with GTCS and 30 age- and gender-matched healthy controls. The patients showed GM volume reduction and a fALFF increase in the thalamus, hippocampus, temporal and other deep nuclei. A significant decrease of fALFF was mainly found in the default mode network (DMN). In addition, epileptic duration was significantly negatively related to the GM volumes and significantly positively related to the fALFF value of right thalamus. A support vector machine (SVM) applied to the GM volume of the right thalamus correctly identified epileptic children with a statistically significant accuracy of 74.42% (*P* < 0.002). A SVM applied to the fALFF of the right thalamus correctly identified epileptic children with a statistically significant accuracy of 83.72% (*P* < 0.002). The consistent neuroimaging results indicated that the right thalamus plays an important role in reflecting the chronic damaging effect of GTCS epilepsy in children. The length of time of a child's epileptic history was correlated with greater GM volume reduction and a fALFF increase in the right thalamus. GM volumes and fALFF values in the right thalamus can identify children with GTCS from the healthy controls with high accuracy and at an individual subject level. These results are likely to be valuable in explaining the clinical problems and understanding the brain abnormalities underlying this disorder.

## Introduction

Idiopathic generalized epilepsy (IGE) with generalized tonic-clonic seizures (GTCS) is a group of epileptic syndromes characterized by the typical seizure symptoms of muscle rigidity, violent muscle contractions of the entire body, body dorsiflexion or bending forward and loss of consciousness. Previous studies have shown that people with this type of epilepsy have significant emotional and behavioral problems (1). These patients have a risk of cognitive limitations and academic difficulties, especially children (2). Exploring the pathophysiological mechanisms of GTCS can provide useful information to guide the clinical diagnosis and treatment of patients with the disease. Although, by concept, routine MRIs are usually normal in IGE with GTCS, the development of highly sensitive neuroimaging techniques has allowed for the identification of subtle functional and structural abnormalities.

Therefore, in recent years, potential etiologies of epilepsy have been explored, in part, by neuroimaging. Neuroimaging techniques have the potential to significantly increase our understanding of the basis of cognitive and behavioral problems in individuals with epilepsy (3–5). Previous studies using functional MRI have shown the widespread activation or deactivation in IGE adults with GTCS (6–8). Patients showed bilaterally and symmetrically altered regional synchronization in the cortical and subcortical structures (8). Studies based on resting-state functional connectivity methods were also used to further explore the neural mechanism of seizure generalization (9–13). Using these functional neuroimaging methods, epileptic circuits can be identified. Abnormal activity and functional connections in the thalamic-cortical network were the most common findings in adults with GTCS in these previous studies. The interictal discharge in the epileptic region would cause BOLD activation to increase in the thalamus, the frontomesial cortex, and the cerebellum and BOLD deactivation in default mode areas (7). The adults with GTCS showed dynamic functional network connectivity and significantly reduced rich club connectivity of the brain (14, 15). Epilepsy effects in adults were detected not only in the brain function but also in the brain anatomical structure. Diffusion tensor imaging examinations have revealed alterations in white matter (WM) tract integrity (9, 16). In addition, recent studies using quantitative structural MRI have shown morphological changes in patients with GTCS (17, 18). Significant decreased gray matter (GM) volume was found in bilateral thalami, the frontal lobe, the insula and the cerebellum in the adult patient group (11, 17). Taken together, these functional and structural neuroimaging aberrances might reflect the progress of long-term epileptic impairments in the brain.

However, we can see that the vast majority of these previous neuroimaging studies in GTCS have been restricted to adult patients. Only a few studies have used neuroimaging methods to explore the neural mechanism exclusively in children and adolescents. All of these epilepsy studies exclusively in children and adolescents were focused on the subtypes of absence epilepsy (19, 20) or juvenile myoclonic epilepsy (21, 22). No study has used MRI technology to explore neural mechanisms exclusively in children with GTCS only. It is accepted that neuroimaging techniques can be used to detect underlying cerebral lesions that may be causally related to seizure disorders or associated neurodevelopmental impairments in children (5, 23). Additionally, the neuroimaging results have been inconsistent regarding brain dysfunction and structural abnormality in these previous studies. For example, thalamus volumes in adult patients with GTCS have been shown to be decreased (17, 24, 25) or normal (26). Therefore, we believe that it is necessary and worth exploring the neural mechanism underlying this type of epilepsy in children to establish its etiology and to plan appropriate clinical care.

In the present study, we focused our attention on epileptic children with GTCS. The multimodel neuroimaging technique was used to examine subtle structural and functional abnormalities in detail. To explore this issue, we took a developed voxel-based morphometry (VBM) tool with diffeomorphic anatomical registration through exponentiated lie algebra (DARTEL) to analyze the structural MRI image (27). This VBM toolbox can minimize the noise level of the segmentation. A previous study has shown that the DARTEL toolbox can achieve much more accurate registration than the previous standard VBM (28). We also examined the difference in the brain's spontaneous activity between the children with GTCS and the normal controls using the resting-state functional MRI. Furthermore, we used a machine learning approach based on the above results to distinguish between groups of subjects. Based on the previous epilepsy studies listed above, we hypothesized that children with GTCS would have structural and functional abnormalities of the brain's thalamic-cortical network compared to the normal controls, which could predict that the structural and functional patterns underlying this type of epilepsy in children may be influenced.

## Methods

### Subjects

Fourteen children with GTCS (all right-handed, four females, mean age: 54.36 ± 38.93 months) participated in this study. All patients were diagnosed with GTCS. The diagnosis of the seizure focus was determined by a comprehensive evaluation, including detailed history and video-EEG telemetry (23 channels). The video-EEG was used to monitor the children for about 24 h, including both the sleep and awake state. The inclusion criteria were as follows: (1) typical clinical symptoms of GTCS, such as tic of limbs followed by a clonic phase of rhythmic jerking of the extremities, loss of consciousness during seizures and no partial seizures; (2) a specific pattern of electrophysiological activity measured by EEG in which generalized spike-and-wave or poly-spike-wave discharges were recorded; (3) no focal abnormality in routine structural MRI examinations. All demographic and clinical information is detailed in Table 1. Out of these 14 patients, the possible epileptogenic focus was determined by combining the clinical features and EEG signals: 5 patients located in the left hippocampus, 2 patients located in the left temporal pole, 2 patients located in the left parietal lobe/angular gyrus, 1 patient located in the right temporal pole, 4 patients without clear origin. The image data of the patient with an origin in the right hemisphere was flipped around the midsagittal plane at the beginning of the analysis, thereby lateralizing the possible epileptogenic focus to the left hemisphere. All child patients were treated with one or two antiepileptic drugs to control seizures. The anti-epileptic drugs used in the patients group included valproic acid, topiramate, levetiracetam. All patients were seizure-free for at least 2 days prior to MRI examination. Thirty healthy controls (all right-handed, 18 females, mean age: 61.17 ± 26.20 months) were also recruited. These control subjects had no history of neurological disorders or psychiatric illnesses. All participants under the age of four were sedated with 10% chloral hydrate during the MRI scanning.

**Table 1:** Demographic and clinical information data of the subjects.

**Characteristics**	**Patients group**		**Controls group**	
	**3D (*n* = 14) Mean ± *SD***	**Rest (*n* = 13) Mean ± *SD***	**3D (*n* = 29) Mean ± *SD***	**Rest (*n* = 30) Mean ± *SD***
Gender (male/female)	10/4	10/3	12/17	12/18
Age (month)	54.36 ± 38.93	50.69 ± 29.13	61.28 ± 26.66	61.17 ± 26.20
Handedness (right/left)	14/0	13/0	29/0	30/0
Seizure onset time (month)	25.54 ± 32.55	22.42 ± 25.3	\	\
Duration (month)	28.89 ± 23.06	28.32 ± 23.93	\	\
Whole GM (mm^3^) ^*^	749.85 ± 58.14		658.37 ± 149.79	
Whole WM (mm^3^)	390.33 ± 70.52		351.20 ± 131.99	
Whole CSF (mm^3^)	136.94 ± 25.10		155.80 ± 46.01	
TIV (mm^3^)	1277.11 ± 123.55		1165.37 ± 281.57	

* *Indicated the whole GM volume between two groups were different significantly*.

Before the image data were collected, we explained the purpose of the study, the study procedures, the possible risks and discomforts to the subject and their parents. Then, research consent forms were signed by the parents of all participants. This study was carried out in accordance with the Declaration of Helsinki and was approved by the Ethics Committee of the Shenzhen Children's Hospital.

### MR Data Acquisition

Anatomical MRI scans and resting-state fMRI scans were acquired on a 3T Siemens scanner (MAGNETOM Trio Tim, Siemens, Germany) using an eight-channel head coil at the Shenzhen Children's Hospital, Guangdong, China. Foam cushions were used in the scanning process to reduce head translation movement and rotation. High-resolution T1-weighted 3D MPRAGE images were acquired for almost all subjects except one control subject: TR/TE = 2,300/2.26 ms, FOV = 200 × 256 mm^2^, 160 slices, 1 mm slice thickness, flip angle = 8°. Resting blood oxygen level-dependent data were acquired in each subject (expect one patient) using an echo-planar imaging sequence with the following scan parameters: TR/TE = 2,000/30 ms, FOV = 220 × 220 mm^2^, matrix = 94 × 94, flip angle = 90°, slice thickness = 3 mm, 36 interleaved axial slices, and 130 volumes. All participants were instructed to lie still with their eyes closed while remaining awake. All acquisitions were visually inspected for imaging artifacts, and none of the participants were excluded on this basis.

### T1-Weighted Data Preprocessing and Statistical Analysis

Structural MRI scans were preprocessed using VBM8 in SPM8 (Wellcome Department of Imaging Neuroscience, London, UK) to gain voxel-wise comparisons of gray matter (GM) volume. The following processing steps were completed: each T1-weighted structural scan was bias-corrected, tissue classified, and registered using linear (12-parameter affine) and non-linear transformations (warping) within a unified model. Individual anatomic scans were partitioned into GM, WM and cerebrospinal fluid segments. As a second step, the GM and WM segments were inputted into DARTEL in order to create a customized DARTEL template. DARTEL was then used to register each structural image to this custom, study-specific template to obtain the individual deformation fields. These individual tissue deformations were then used to warp and modulate each participant's GM and WM tissue maps for non-linear effects. The modulated GM and WM were written with an isotropic voxel resolution of 1.5 mm. Finally, those modulated GM images were smoothed with a full-width half-maximum kernel of 6 mm. After completing these image analyses, we obtained smoothed modulated GM images to be used for statistical analysis.

We used SPM8 for all statistical analyses. Voxel-wise GM volume differences between the children with GTCS and the normal controls were examined using a two-sample *t*-test. Additionally, an absolute threshold mask of 0.1 was used to avoid possible edge effects between GM and WM. All findings were considered significant at a voxel level of *p* < 0.05 (FDR corrected, cluster size > 15). Age, gender and total intracranial volume (TIV) were added as additional covariates in all these analyses above, which means that all effects could be explained by age, sex, and TIV differences were removed from the data. Whole-brain GM volume, WM volume, cerebrospinal fluid (CSF) volume, and TIV were extracted and compared between both groups.

To test whether the patient's clinical characteristics had some effect on the brain's structural changes, the correlation between local GM and the duration of epilepsy were analyzed. Correlation analyses were calculated at each voxel within a mask obtained from two-sample *t*-tests between the two groups. Age, gender, and TIV were added as additional covariates in the correlation analysis. For the correlation analysis, the statistical map was set at a significance level of *p* < 0.001, uncorrected for multiple comparisons, with an extended threshold looking for clusters with at least 15 contiguous voxels. The clusters showing significant correlation were identified as core regions reflecting the damaging effect from epilepsy. In the following resting-state fMRI analyses, we want to identify whether there are functional abnormalities and significant correlation with epilepsy duration in these areas.

### Resting-State Data Preprocessing and Statistical Analysis

The resting-state fMRI data was processed using Data Processing Assistant for Resting-State fMRI (DPARSF) software (http://www.restfmri.net) (29). The first 10 functional images per subject were excluded from the analysis to ensure magnetization equilibrium. Then, the fMRI data were slice-timed with a reference point at the median image and realigned to the first image. No translation or rotation parameters in any given data set exceeded ±2 mm or ±2°. Afterward, all the realigned images were spatially normalized into standard Montreal Neurological Institute space using the DARTEL template and spatially smoothed with a 6-mm full width at half maximum Gaussian kernel. Finally, an improved approach of the amplitude of the low-frequency fluctuation (ALFF) method, fractional ALFF (fALFF), was used for detecting a regional signal change of spontaneous activity (30).

The fALFF analysis was performed by REST software (www.restfmri.net). Linear trends were removed before the time course data from each voxel. Then the data was subjected to fast Fourier transformations into the frequency domain. The fALFF was computed as the ratio of the power spectrum of low-frequency (0.01–0.08 Hz) to that of the entire frequency range. The fALFF for each voxel was normalized and compared between the two groups using the two-sample *t*-test. A threshold for significance was set at corrected *p* < 0.05 (combined height threshold *p* < 0.001 and a minimum cluster size of 13 voxels) by employing the Monte Carlo simulations using the AlphaSim program in the REST software. Age and gender were controlled as covariates in all the above statistical analyses.

We compared the structural and functional analysis results. The significant clusters of fALFF analyses results, which showed the location overly or nearby the GM volume correlation results, were considered to be a region of interest. The mean fALFF of each region of interest was extracted and normalized with Fisher's z-transformed function. The correlations between the transformed fALFF value and the epilepsy duration were calculated.

### Support Vector Machine Analysis

The above analyses were all based on group-level statistics. However, the need of clinical practice is to classify or evaluate patients at the individual level. These traditional approaches do not permit an evaluation of the discriminative power of analysis results at the individual level. In recent years, the multivariate pattern analysis approach has been used in neuroimaging data to extract patterns and to categorize individual observations into different categories (31, 32). In the present study, we used a specific multivariate pattern analysis approach known as support vector machine analysis (SVM). SVM was implemented using PRoNTo (Pattern Recognition for Neuroimaging Toolbox) software (http://www.mlnl.cs.ucl.ac.uk/pronto/) version 2.0. Individual T1-weighted images were treated as points located in a high dimensional space defined by the GM volumes in the preprocessed images. In order to ensure that the SVM models were based on the same set of voxels, we selected the cluster showing significant correlation between the GM volume and the epilepsy duration as a mask. In the case of the structure MRI data, this mask was applied to each image to select the GM volume as a feature in the modeling. The classifier considered in the present work (i.e., epilepsy patients vs. healthy controls) is based on binary SVM machines. During the cross-validation step, a “leave-one-subject-out” method was used (31, 33). The data were split to a training set consisting of all the samples from all but one subject and a validation set consisting of the samples from the left-out subject. In order to assess the overall accuracy of the SVM, this procedure was repeated for each subject pair. The classification procedure was repeated 1,000 times. Statistical significance for classification accuracy was determined by permutation testing.

Similar SVM analysis was used on the fALFF value of resting-state fMRI scans. The cluster showed a significant correlation between the transformed fALFF value and the epilepsy duration, which was extracted as a mask in the SVM model. In the case of the resting-state fMRI data, this mask was applied to each preprocessed image to select the fALFF value as a feature in the modeling. SVM classifier machines and the “leave-one-subject-out” cross-validation method were used. The classification procedure was repeated 1,000 times. Statistical significance for classification accuracy was determined by permutation testing.

## Results

### Between-Group VBM Analyses of GM Volume

**Figure 1:**
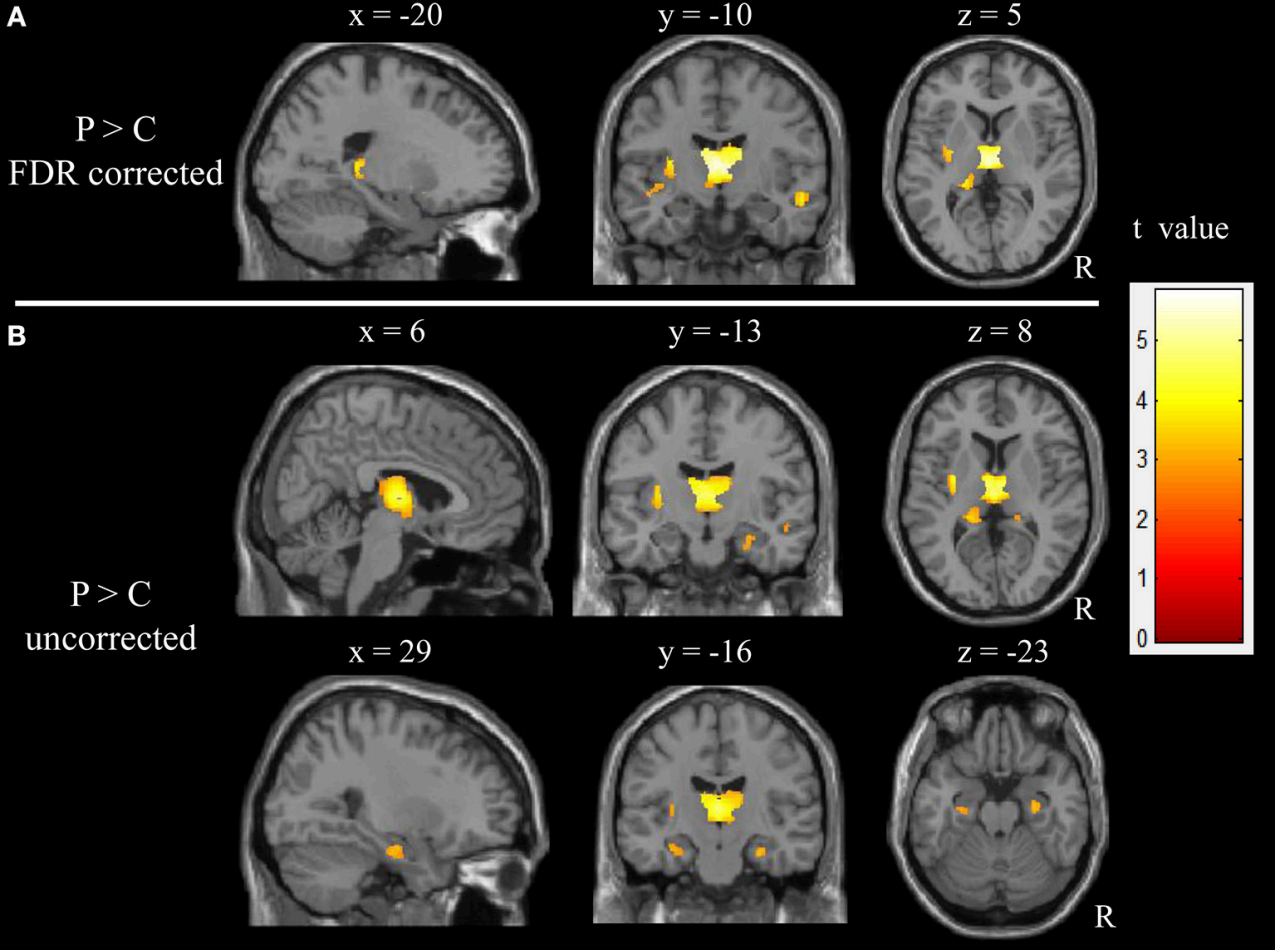
Significant changes of GM volume in children with epilepsy. **(A)** The threshold of whole brain: *p* < 0.05, FDR corrected, cluster size > 15; **(B)** The threshold of whole brain: *p* < 0.001, uncorrected, cluster size > 15. The color intensity represents T-statistic values at the voxel level. P, patients; C, normal controls; R, right hemisphere.

The results obtained from the two-sample *t*-test showed a significant difference in the GM volume between two groups (FDR corrected; Figure 1 and Table 2). Compared with healthy children, those with GTCS showed decreased GM volume in the bilateral thalamus, superior temporal gyrus (STG), left putamen, insula, hippocampus and gyrus rectus. When the statistical threshold was broadened to a voxel level of uncorrected *p* < 0.001 with a cluster size >15, children with GTCS showed decreased GM volume in the right hippocampus. No significant increased GM volumes were found in the patient group. Compared with the controls, the children with GTCS showed a significant decrease of whole-brain GM volume (see Table 1). No significant difference was found in the whole-brain WM volume, whole-brain CSF, or TIV.

**Table 2:** Regions of significant volume changes between patients and controls.

**Comparisons**	**Statistical values**			**Coordinates anatomical location**			
	**Cluster size**	***t*-value**	***p*-value**	**x**	**y**	**z**	**Region**
Control>Patient	4318	5.78	0.000	−1	−15	9	L Thalamus
		5.40	0.000	8	−9	6	R Thalamus
		5.01	0.000	−11	−10	15	L Thalamus
		4.11	0.000	−16	−36	8	L Hippocampus
	183	4.05	0.000	−29	−7	4	L Putamen
		3.47	0.001	−34	−8	7	L Insula^*^
	19	3.51	0.001	30	−13	−20	R Hippocampus^*^
	157	3.97	0.000	−5	24	−26	L Rectus
	170	3.79	0.000	56	−9	−12	R Sup temporal gyrus
	44	3.76	0.000	−45	−10	−12	L Sup temporal gyrus
		3.64	0.000	−38	−12	−5	L Insula
Patient>Control	no						

*The MNI coordinates and t-values for the local maxima of the centers of the voxel clusters. R, right hemisphere; L, left hemisphere; Sup, superior. Threshold for significant clusters reported here was set at p < 0.05, FDR correction, cluster size of 15*.

* *Indicated the cluster is significant at p < 0.001, uncorrected, cluster size > 15*.

### Relationships Between Local GM and Epilepsy Duration

**Figure 2:**
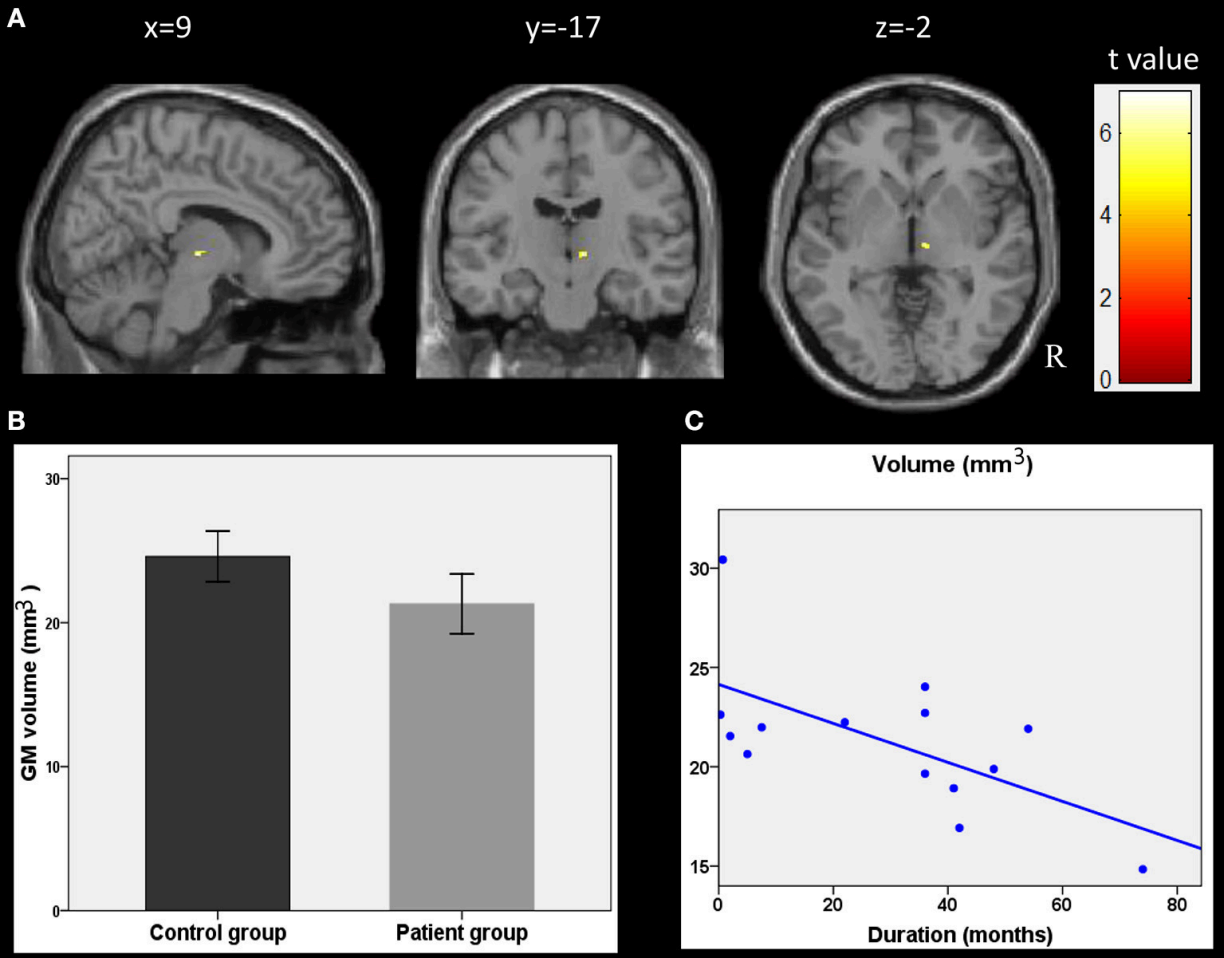
Correlations between GM volumes and clinical characteristics. **(A)** Results of the correlation analysis between the epilepsy duration and GM volumes in children with epilepsy (*p* < 0.001, uncorrected, cluster size > 15). **(B)** GM volumes in the cluster of right thalamus as showing in plane A. **(C)** the scatter plots between these cluster volumes and the epilepsy duration. The line was also drawn, which represent the direction of the association. R, right hemisphere.

The correlation analyses showed that the duration of epilepsy was substantially negatively correlated with the regional GM volume of the right thalamus (*t* = 6.96, *p* < 0.001, cluster size = 15; Figure 2A). To visualize the correlation results, we extracted the GM volumes of the right thalamus (Figure 2B). We then pointed the scatter plots between the volumes of this cluster and the duration of epilepsy (Figure 2C). A line was drawn, which represented the direction of the association. The right thalamus was considered to be the core region reflecting the damaging effect from epilepsy.

### Between-Group fALFF Differences

**Figure 3:**
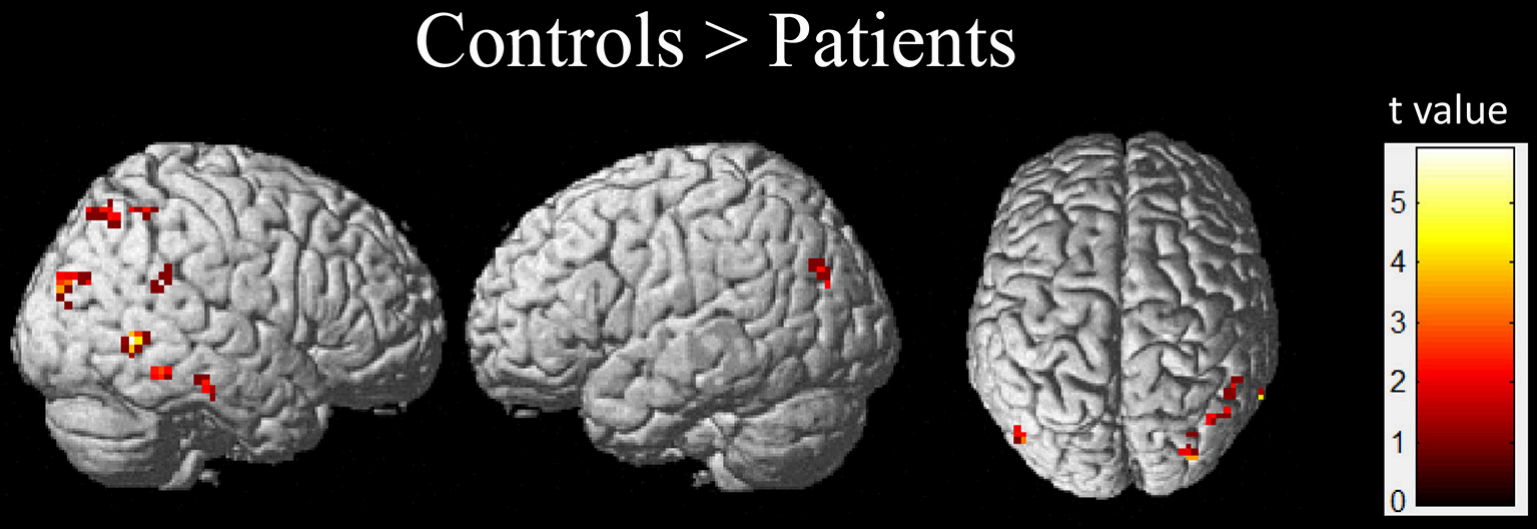
The regions showed the significant decrease of fALFF in the children with GTCS.

**Figure 4:**
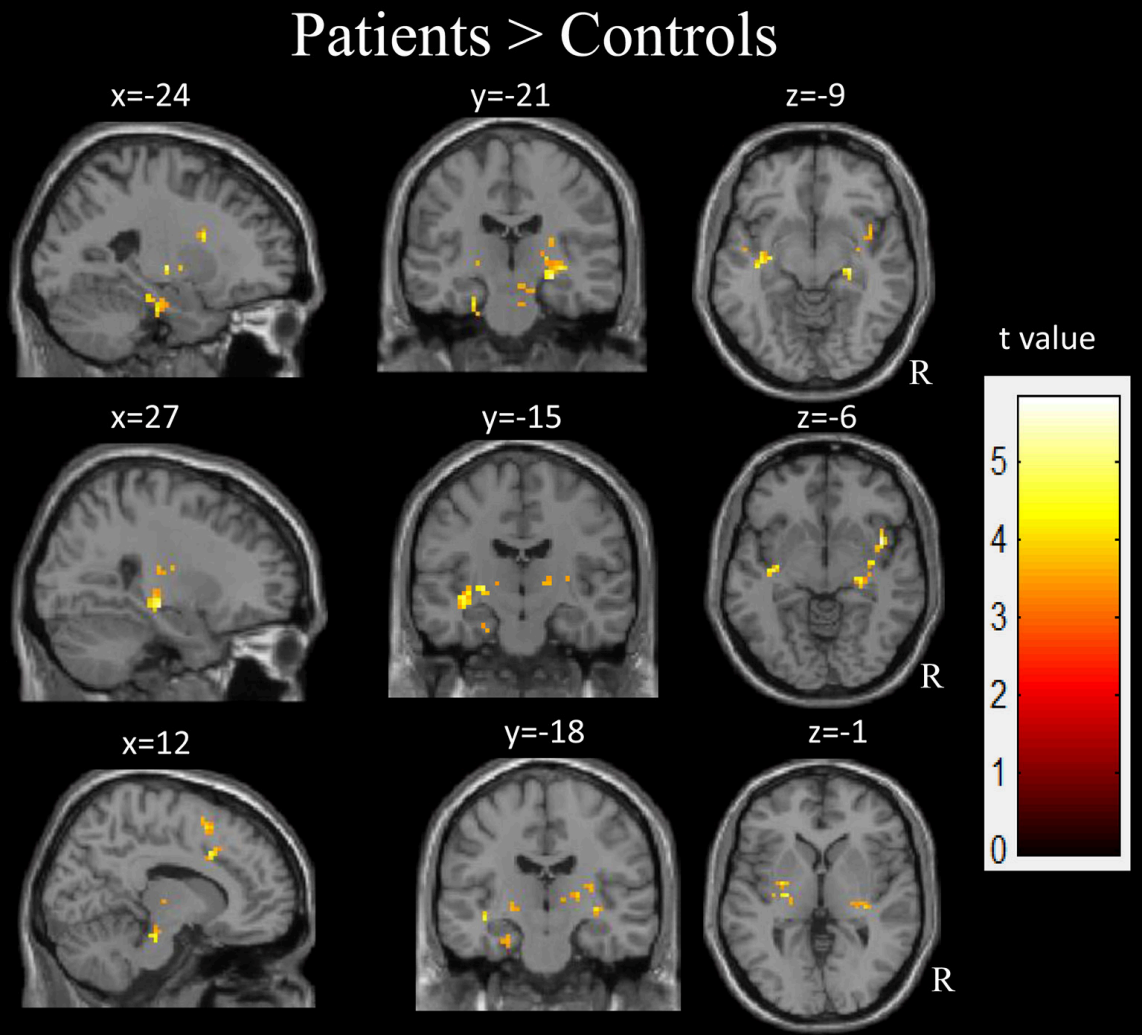
The regions showed the significant increase of fALFF in the children with GTCS. R, right hemisphere.

Compared with the healthy controls, the children with GTCS showed decreased fALFF in the right angular, right middle temporal gyrus (MTG) and middle occipital gyrus (AlphaSim correlation, Table 3, Figure 3). While the statistical threshold was broadened to a voxel level of uncorrected *p* < 0.001 with cluster size > 8, children with GTCS also showed decreased fALFF in the bilateral angular, right inferior parietal lobule and inferior temporal gyrus (ITG). In addition, the patient group showed increased fALFF in some regions of the right hemisphere, such as the hippocampus, thalamus, supplementary motor area (SMA), insula, and brain stem (AlphaSim correlation, Figure 4, Table 3). The fALFF in the left parahippocampus, left MTG and STG also showed a significant increase in the patient group (AlphaSim correlation). While the statistical threshold was broadened to a voxel level of uncorrected *p* < 0.001 with cluster size > 8, the left thalamus and left superior frontal gyrus (SFG) also showed increased fALFF in the children with GTCS related to the controls.

**Table 3:** Summary of significant activations between patient and control from the whole-brain analysis.

**Comparisons**	**Statistical values**			**Coordinates anatomical location**			
	**Cluster size**	***t-*value**	***p*-value**	**x**	**y**	**z**	**Region**
**CONTROL>PATIENT**							
	21	5.89	0.000	60	−57	0	R Mid temporal gyrus
	9	4.66	0.000	63	−24	−21	R Inf temporal gyrus^*^
	11	4.95	0.000	60	−57	0	R Inf temporal gyrus^*^
	22	4.50	0.000	33	−87	24	R Mid occipital gyrus
	18	4.06	0.000	42	−69	54	R Angular
	10	3.80	0.000	48	−45	21	R Angular^*^
	10	3.92	0.000	−48	−75	30	L Angular^*^
	10	4.15	0.000	51	−54	54	R Inf parietal lobule^*^
**PATIENT>CONTROL**							
	19	4.30	0.000	−24	−21	−27	L ParaHippocampus
	45	4.68	0.000	−36	−15	−6	L Mid temporal gyrus
		3.74	0.000	−51	−6	−12	L Sup temporal gyrus
	50	5.11	0.000	27	−21	−9	R Hippocampus
		3.86	0.000	21	−18	6	R Thalamus
	8	4.67	0.000	−24	−15	0	L Thalamus^*^
	8	4.49	0.000	−15	−12	3	L Thalamus^*^
	70	4.51	0.000	11	12	57	R Supplementary motor area
	19	4.46	0.000	12	−24	−21	R Brain stem
	16	5.77	0.000	42	6	−6	R Insula
	10	4.01	0.000	−15	12	42	L Sup frontal gyrus^*^

*The MNI coordinates and t-values for the local maxima of the centers of the voxel clusters; R, right hemisphere; L, left hemisphere; Inf, inferior; Sup, superior; Mid, middle. Threshold for significant clusters reported here was set at p < 0.05 (combined height threshold p < 0.001 and a minimum cluster size of 13 voxels) using AlphaSim correction*.

* *Indicated the clusters significant at the threshold of p < 0.001 (uncorrected), cluster size > 8*.

### Correlation Between fALFF and Epilepsy Duration

**Figure 5:**
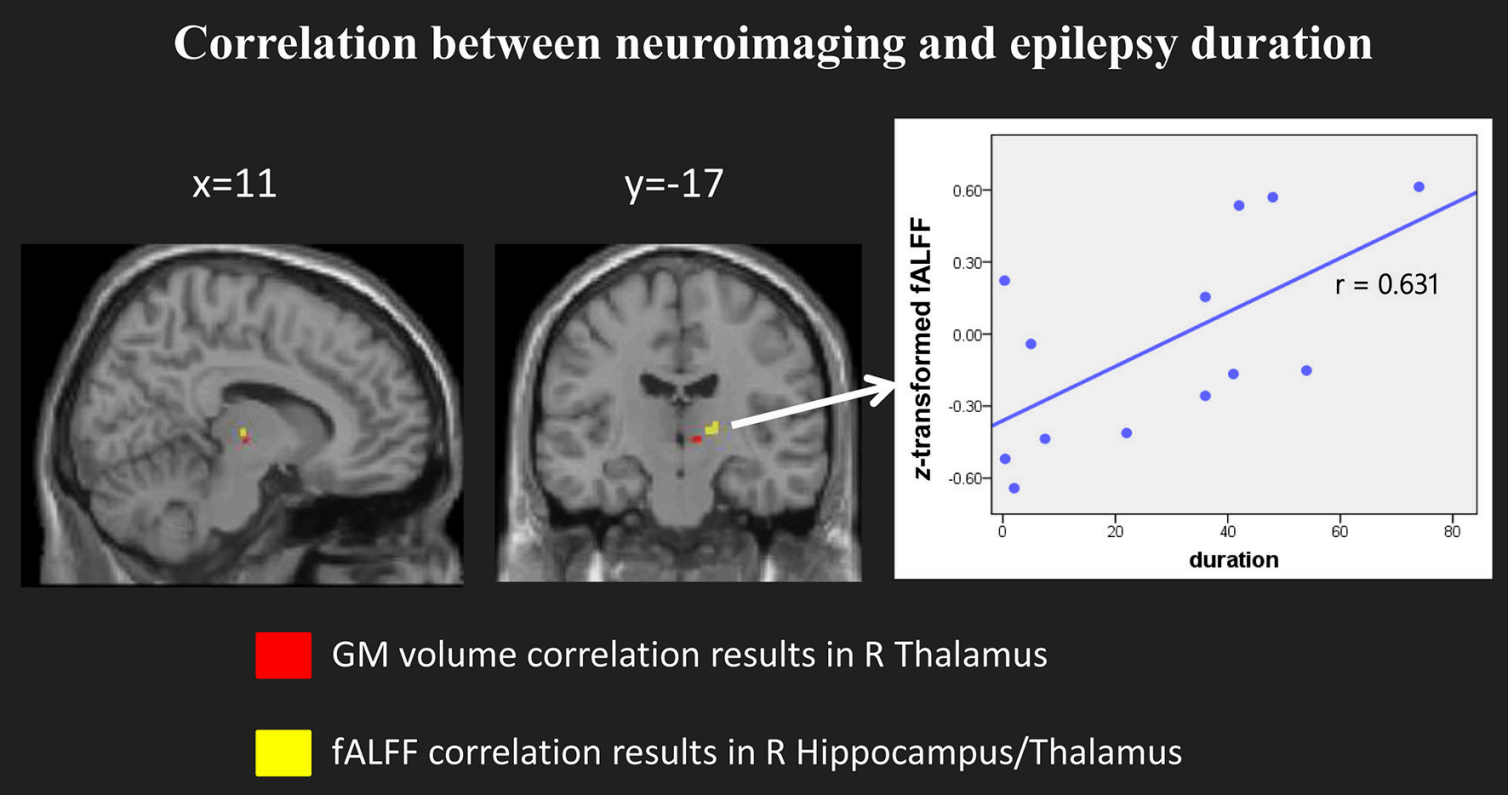
Significant correlation between the fALFF and epilepsy duration. **(Left)** panel showed the location of the clusters with significant correlation in both model imaging. **(Right)** panel showed the scatter plots and the direction between the normalized fALFF of right hippocampus/thalamus and epilepsy duration.

When we compared the structural and functional results, we found the location of right hippocampus/thalamus of fALFF results partly overlapped with the GM volume correlation results (see Figure 5, left panel). We then selected the right hippocampus/thalamus cluster as our region of interest and extracted the mean fALFF value of this cluster. Then we normalized the fALFF data and calculated the correlation between normalized fALFF and epilepsy duration. There was a significant positive correlation between the normalized fALFF and epilepsy duration (*r* = 0.631, *p* = 0.021, see Figure 5, right panel).

### SVM Classification Results

**Figure 6:**
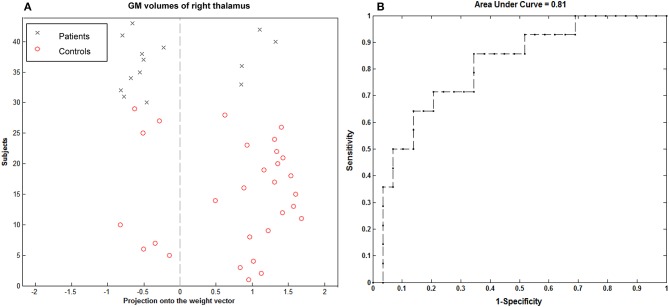
Classification plot **(A)** and receiver operating characteristic curve **(B)** for the comparison between 14 children with GTCS and 29 healthy controls using GM volume from right thalamus of T1-weighted images. This yielded a total accuracy of 74.42% (75.86% sensitivity, 71.43% specificity), statistically significant at *P* < 0.002.

**Figure 7:**
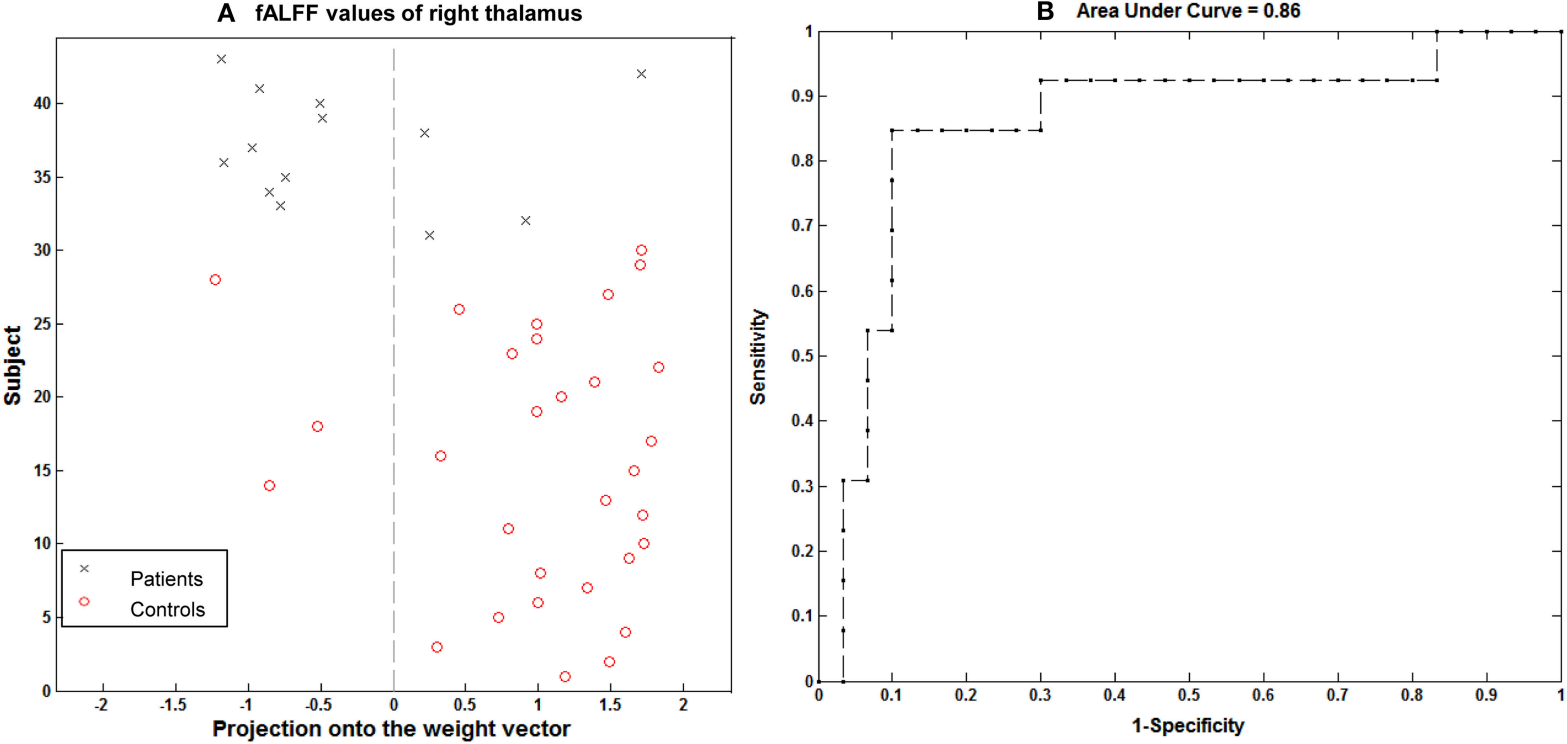
Classification plot **(A)** and receiver operating characteristic curve **(B)** for the comparison between 13 children with GTCS and 30 healthy controls using fALFF from right thalamus of resting-state fMRI. This yielded a total accuracy of 83.72% (90% sensitivity, 69.23% specificity), statistically significant at *P* < 0.002.

Using the imaging feature, GM volume in the right thalamus was shown to be significantly correlated with epilepsy duration; the analysis of SVM classification achieved a classification accuracy of 74.42%, statistically significant at *P* < 0.002 (Figure 6). This overall classification accuracy of the algorithm measures its ability to correctly classify an individual as epileptic or part of the control. The sensitivity was 75.86% and the specificity was 71.43%. Using the imaging feature of the fALFF value in the right hippocampus/thalamus cluster, it was shown to be significantly correlated with the epilepsy duration; the analysis of SVM classification achieved a classification accuracy of 83.72%, statistically significant at *P* < 0.002 (Figure 7). This yielded a sensitivity of 90% and a specificity of 69.23%.

## Discussion

In the present study, structural and functional MRI were combined to explore the abnormalities of GM volume and spontaneous neuronal activity in pediatric IGE patients with GTCS. We performed volumetric measurements with the VBM method and explored the associations between volumetric and clinical characteristics. The fALFF were calculated from the functional MRI to measure the resting-state functional abnormalities in GTCS. The main findings of the present study were consistent with our predictions. First, compared with the normal controls, the patients had significant volume reductions in the bilateral thalamus and STG. The GM volume in other deep nuclei, such as left putamen, insula and bilateral hippocampus, also showed a significant decrease in the patient group. Second, the children with GTCS showed both an increase and decrease of fALFF value in the brain compared to the controls. Significant increases of fALFF for patients were found in the bilateral hippocampus, bilateral thalamus, right insula, right SMA, left SFG, left STG, and MTG. A significant decrease of fALFF for patients was found in the main regions of the default mode network (DMN), including the bilateral angular, right middle occipital gyrus, right ITG and MTG. Thirdly, the correlation analyses indicated that the epileptic duration was negatively related to the volume reduction in the right thalamus and positively related to the fALFF value in the right hippocampus/thalamus. We also demonstrated that child patients with GTCS can be distinguished from healthy controls using GM volumes and fALFF values from right thalamus at the level of the individual. The functional and structural analysis results were well-consistent with each other. This supports our hypothesis that GTCS can induce abnormal function and structure in these epilepsy-related areas. These results may have important implications for understanding brain abnormalities and the clinical characteristic of this disorder in children.

### Significant Decrease of GM Volume in the Children With GTCS

The thalamus plays a multifunctional role in the initiation, propagation and inhibition of epileptic activity via cortical-thalamic-subcortical circuits (34). Previous morphological MRI studies in adults with GTCS have demonstrated GM structural abnormality in the bilateral thalamus and cortical structures (11, 17). A significant decrease of GM volume in the bilateral thalamus was the usual neuroimaging characteristic in adults with GTCS. In the present study, the children with GTCS also showed a significant GM volume decrease in the bilateral thalamus. The structural abnormalities in the bilateral thalamus in the present study are in line with the above-mentioned adult MRI studies (11, 17, 24, 25, 35). Our study further implied that the structural alterations of the bilateral thalamus were common results in both adults and children with GTCS. In addition, this main structure abnormality in the bilateral thalamus of the children with GTCS can also be explained by the functional role of thalamus. Abnormal thalamic function is a common phenomenon in this type of epilepsy. Previous functional MRI studies have shown that brain activation (8) and functional connectivity (10, 11, 13) with other regions in the thalamus changed significantly in patients with GTCS. Abnormal cortical-subcortical electrical discharges were transferred through the thalamus to generate tonic seizure activity and cognitive impairment. These functional neuroimaging studies demonstrated that thalamocortical circuits are necessary for the generation of typical seizures (8). The bilateral thalamic structural abnormalities in the present study, combined with these previous adult studies, may provide the physical basis of the abnormal thalamic function.

Although the phenomenon of thalamic structural abnormalities was found in the present pediatric cohort as well as in most previous studies of adult patients with GTCS (17, 25, 35), other previous studies also reported no significant difference compared to the controls (26, 36). The main reason underlying these conflicting results might be the different analysis technologies used across these studies. In ours and other recent studies, a customized VBM with DARTEL method was used to explore the subtle structural differences between the two groups' subjects (37, 38). This new method was developed with a unified segmentation model and a high-dimensional normalization protocol. The accuracy of segmentation and normalization in this new method was improved more significantly than the previous standard VBM (28). Additionally, the participants in the present study were all children. During the analysis processing, a systematic bias into the segmentation routine would have been introduced if we used the standard adult reference template. To avoid this bias, the DARTEL toolbox was used to create a study-specific template. This analysis method would in some respects improve the accuracy of our results.

In addition to the bilateral thalamus, decreased GM volumes were also detected in the left insula, putamen and bilateral hippocampus of children with GTCS. The insula is a region of convergence of multisensory inputs, with strong connections to the thalamus and several other cortical areas (39). Impairment in the insula would affect motor and somatosensory functions. A previous neuroimaging study in adult IGE with GTCS only showed an increased regional synchronization in the insular cortex (8). These motor manifestations would be the result of the functional impairment in the insula. Insula impairment with GM volume decrease has also been found in adults with GTCS (17) and juvenile myoclonic epilepsy (40). In the present study, the decreased GM volume detected in the left insula was consistent with these previous studies, which implied that the structural impairment of the left insula was also present for children with GTCS. The putamen is a round structure located at the base of the forebrain. The main function of the putamen is to regulate movements and influence various types of learning. Anatomical evidence has shown that the putamen is interconnected with the cerebrum, thalamus, and brainstem. A VBM study on adult IGE patients with GTCS found significant GM volume decreases in the thalamus and putamen (24). These previous functional neuroimaging studies have shown that brain activity increased in the thalamus, putamen, insula and cerebellum of adults and children with IGE (41, 42). In the present study, the structural impairment in the putamen of children confirmed these previous studies and implied that this region was involved in the communication processing of epileptic seizures in children with GTCS. Critical memory-related structures, such as the hippocampus, showed a significant loss of GM volume. Additionally, the result of the left hippocampal GM volume decrease can stand the correction. This result is consistent with a previous study (18) and confirmed that the left hippocampus is more vulnerable to GTCS than the right hippocampus. Combined with these previous studies, subtle structural alterations in the thalamus, putamen insula, and hippocampus of our results implied that brain anatomy architecture was disrupted in children with GTCS. All these regions are sensitive to the pathophysiology of GTCS in children. At the molecular level, several mechanisms can explain these anatomical changes, such as changes in cell size and neuronal or glial atrophy (43). Therefore, the clinical characteristic of children with epilepsy may be a response to structural impairments in the thalamus, insula, putamen, and hippocampus.

GM volumes in the bilateral STG of the patient group also decreased significantly. A similar result was found only in a previous epilepsy study (24). Recent VBM analyses of epileptic patients with GTCS did not find structural abnormalities in the STG. The reason for this difference between our study and the previous one might be that the participants in the present study were all children. Our VBM results in children with GTCS were, to some extent, different from the VBM results of adults. Structural changes of the frontal lobe and cerebellum were usually detected in previous adult studies (17, 35), which were not found in the present study. Whether, the participant's age is the only reason for this difference warrants further research. Additionally, we detected a significant increase of whole-brain GM volume in the patient group. This result is not consistent with our expectation. We expected that the whole brain GM volume difference between two groups would not be significant. The reasoning behind this result may be that the gender is mismatched between two groups. Future studies with matched gender between the two groups would eliminate this and provide a check on our inference.

### Significant Difference of Brain Spontaneous Activity Between the Patients and the Controls

We also used the fALFF value to measure the proportional decrease of BOLD fluctuations in the low frequency band. The children with GTCS showed increased fALFF mainly in the bilateral thalamus, right parahippocampus, right insula, left hippocampus, left SFG, left MTG, left STG, and decreased fALFF in the bilateral angular, right ITG and MTG. The fALFF value is thought to reflect spontaneous activity underlying stable states and functional connections between distinct brain regions. Increased fALFF values in the bilateral thalamus of the children with GTCS are concordant with the findings revealed by previous adult fMRI studies (7, 8, 12, 42). These previous adult epilepsy studies found that the patients with GTCS showed highly synchronized activity in the bilateral thalamocortical network (7, 8, 13). Brain activity and local coherence increased in the bilateral thalamus, reflecting functional abnormalities in this region. The thalamus plays a crucial and functional role in the initiation, propagation, and inhibition of epilepsy activity via cortical-subcortical networks. Previous studies have found that one region showing higher energy demands of brain communication may be more vulnerable to deficits in energy delivery or utilization (44). A significant increase of fALFF in the thalamus implied that the function of this region was disrupted. Abnormally high energy in this region could render a disruption of information communication. So that the patients with GTCS showed tonic seizure activity and cognitive impairment. In the present study, fALFF values of the bilateral thalamus showed a significant increase at rest, which provides initial evidence of functional abnormalities in the bilateral thalamus of children with GTCS. Additionally, our functional and structural analysis results from the bilateral thalamus were well-consistent with each other. This important result of the present study indicates that structural and functional abnormalities in the bilateral thalamus represent the main neuroimaging characteristic for children with GTCS.

Group differences with significant increases of fALFF were also observed in the bilateral hippocampus, right insula, SMA, left superior frontal gyrus, left superior and middle temporal gyrus. All these regions showed the children with GTCS presented widespread functional changes. However, the increased fALFF results of children with GTCS were not completely consistent with the results from adults with GTCS. One previous study of adults with GTCS found that the patients showed a significant increase of fALFF in the anterior cingulum, bilateral thalamus, putamen, and cerebellum (42). Measurements of the synchronization of spontaneous resting-state fMRI signal oscillations in adults with GTCS have shown that significant increases of regional homogeneity in patients were found bilaterally in the thalamus, cerebellum, brain stem, insula, parahippocampus gyrus, fusiform gyrus, and sensorimotor area (8). Adult patients with IGE showed BOLD activation in the thalamus, the frontomesial cortex, the cerebellum and BOLD deactivation in default mode areas (7). Comparing our results from children with these previous results from adults, we can see that most results were consistent except the bilateral cerebellum. The cerebellum showed significant changes of fALFF and regional homogeneity in previous adult neuroimaging studies. However, our functional and structural imaging results did not find significant changes in fALFF values or GM volumes in the bilateral cerebellum of children with GTCS. This special result may be explained by its functional roles. Previous studies on adults have shown that the cerebellum, insula, and SMA have been implicated in motor function (8, 41). The function of this region was disrupted in adults with IGE. The adults with IGE showed complex motor manifestations during epileptic seizures. Although we did not find significant spontaneous activity changes of the cerebellum in children with GTCS, a significant increase of fALFF in the right insula, right SMA, and left SFG were detected. Previous studies have confirmed that all these regions have an important role in motor function (41). The neuroimaging expression of motor abnormalities in children with GTCS is the increased spontaneous activity in these motor-related regions except the cerebellum. Future studies should focus on this difference between the adults and children with GTCS to detect in which stage the difference in the cerebellum began to appear.

Significantly decreased fALFF in children with GTCS was found in the main areas of DMN, such as the right temporal lobe and the bilateral angular gyrus. This result was consistent with our expectations. Previous epilepsy studies have shown that DMN regions were considered to be related to abnormal functional integration and ictal unconsciousness in generalized epilepsy (45, 46). Abnormal activities in the regions of DMN were also investigated in adult GTCS patients, which might reflect the long-term injurious effects of epileptic action (7, 10, 42, 47). The functional role of the DMN regions was disrupted and the neural activities were restrained. The functional abnormality in DMN also reflected that its information communication with other networks was impaired (12, 14, 15). However, the main decrease in fALFF in the present study was located in the temporal and parietal areas; we did not find significant a decrease of spontaneous activity in the mesial prefrontal cortex as has been shown in previous GTCS studies on adults. Our VBM results also did not find significant changes of GM volumes in the mesial prefrontal cortex. This is another detected difference between the children and adults with GTCS.

Combining structural and functional results in the present study, we can see that common brain changes of the children with GTCS were located at the bilateral thalamus and hippocampus. These significant results of children with GTCS were consistent with the previous imaging studies in adults with GTCS. Thus, we can say that the bilateral thalamus and hippocampus were the GTCS epilepsy-related areas in both adults and children. Additionally, we can say that the children with GTCS did not show significant changes of fALFF in the bilateral cerebellum or the mesial prefrontal cortex. Our imaging results indicated that the children with GTCS showed a different pattern of brain abnormality from the adults with GTCS.

### Correlation Between Neuroimaging Index in the Thalamus and Duration of Epilepsy

More importantly, our results showed a negative correlation between the GM volume in the right thalamus and the seizure duration. The negative correlation with this region in the thalamus was largely in agreement with previous studies (17, 35). This correlation suggests that the GM volume decrease in the thalamus may be due to seizures. A greater thalamus volume decrease appeared in children with a longer history of epilepsy. In addition, the fALFF value in the right hippocampus/thalamus also showed a positive correlation with the epileptic duration in children with GTCS. The longer the child had GTCS, the more of his brain's functional abnormalities were found in the right hippocampus/thalamus. Combining our structural and functional correlation results, we can see that both brain activity and the structure of the right thalamus simultaneously showed significant correlation with epilepsy duration. Progressive fALFF and GM volume change in the right thalamus may be due to the long-term effect of epilepsy. Previous studies on patients with epilepsy have found that the thalamus plays an important role in the subcortical structure in the generalization and transmission of epileptic seizures (7, 8, 48). Our correlation results again confirmed this view and demonstrated that the thalamic volume and resting BOLD fluctuations might have some potential applications for clinical diagnoses in future studies. Additionally, the reason for significant correlation in this study may be the result of an epileptic disease effect. Brain GM volume decreases are a common phenomenon for neurological and psychiatric diseases. Increased brain activity in the thalamus was correlated with the higher energy demands of brain communication after epilepsy. The normal development of the function and the connectivity patterns in the thalamus were modulated with this disease. Therefore, the longer the epileptic history of the child, the more serious their development trajectory changes were detected. As a result, the child with longer GTCS duration showed more structural abnormality and higher brain activity in the thalamus. Significant structural and functional changes of the right thalamus can be considered to neuroimaging characteristics of children with GTCS. Here, we note that the voxel-based correlation results between GM volume and epilepsy duration cannot stand up to the correction. The main reason for this may be that the sample size is too small and the distribution of epilepsy duration is asymmetrical (Figure 2C). If the sample size and the distribution of epilepsy duration are adapted in future studies, the voxel-based correlation results would improve.

In the present study, although the significant differences of brain volumes and fALFF values between two groups were found in the bilateral thalamus and hippocampus, significant correlations between the neuroimaging index and epilepsy duration were found only in right thalamus/hippocampus. The reason for this lateralization effect may be that the location of epileptogenic focus was lateral in most of patients. The possible epileptogenic focus of most patients in our present study was located in the left hemisphere. The contralateral epilepsy-related region (such as the right thalamus/hippocampus) is recruited to adapt the brain's functional and structural organization in children with GTCS. Significant correlation results in right thalamus/hippocampus may reflect the chronic damaging effect of GTCS epilepsy. Our machine learning results also verified the above inference and showed that the right thalamus/hippocampus play an important role in brain organization for children with GTCS. This phenomenon has also been detected in other epilepsy studies. A recent neuroimaging study on temporal lobe epilepsy has found that the contralateral parahippocampus white matter bundle showed significant pathology (49). The diffusion characteristics of the contralateral parahippocampus white matter bundle could classify individual patients with persistent postoperative seizure from seizure-free patients. It would be valuable in the future to study this lateral effect of GTCS children with a unilateral epileptogenic focus.

### Reliable Identification of Children With GTCS From the Healthy Controls

In this study, we classified the children with GTCS from the healthy controls by the linear SVM classifier, corresponding to an accuracy of 74.42% based on the GM volume of the right thalamus and of 83.72% based on the fALFF value of the right thalamus. In recent years, multivariate pattern analysis methods have been applied to categorize epilepsy patients from healthy controls (15, 50, 51) as well as in surgery outcome prediction (52). However, all these studies focused on epilepsy in adults. No previous studies have applied a machine learning approach on children with GTCS. In the present study, the right thalamus showed significantly different GM volume and fALFF values between both groups. The correlation between these imaging indexes of the right thalamus and seizure duration are also significant. The significant imaging-index changes of the right thalamus may be considered to be neuroimaging-based biomarkers to differentiate children with GTCS from healthy controls. In order to verify this view, machine learning methods were used. The GM volume and fALFF value in the right thalamus were set as features of the machine model, and the SVM model was used to discriminate between the groups of subjects. The results demonstrate that these abnormalities allow accurate discrimination between children with GTCS and healthy controls at the level of the individual. Combining the GM volume and fALFF changes of the right thalamus can be considered to be neuroimaging-based biomarkers for differentiating children with GTCS from healthy controls at the individual subject level.

During the machine learning processing, the simulated replicability was implemented via the leave-one-subject-out cross-validation approach. Here, our choice for leave-one-subject-out, but not the other cross-validation approach, was determined by our sample size (53). The sample size of our present study was small. If we chose another cross-validation approach, such as 5-fold cross-validation, this would induce a high level of variance (high bias). Using leave-one-subject-out cross-validation is often better when the size of the dataset is small. In addition, previous studies have also shown that this method mirrors the clinically relevant use-case scenario of diagnosis and is therefore especially useful in clinical diagnostic applications (31, 33). Our research objects were children with clinical epilepsy; this is another consideration factor that induced us to select the leave-one-subject-out cross-validation approach. Our analysis results indicated that this model is useful. GM volume and fALFF values of the right thalamus have potential value in clinical diagnostic applications.

### Limitations

There are several limitations associated with our study. First, the sample size used was relatively small. The epileptogenic focus of the patients was diversity. Future studies with larger sample sizes and more homogeneous patients may provide further insights. Second, different antiepileptic medication and sedation were used by the patients. These limitations may have confounded the results. Using a homogeneous group of patients and scanning conditions should be considered in future studies. Third, we did not find significant differences in the cerebellum or the mesial prefrontal cortex of the functional and structural images. Although this region showed a significant difference of fALFF and regional homogeneity between the adults with IGE and the normal controls, the children with GTCS included in the present study did not show significant changes from the normal controls. Our present study cannot give a complete explanation of this result, leading to a need for further study.

## Conclusion

The present study demonstrated that children with GTCS have specific changes in their brain's structural and functional patterns. A significant decrease of GM volume and significant increase of spontaneous activity in the brain were detected in the bilateral thalamus, hippocampus, and other deep nuclei of children with GTCS when compared with the controls. Significantly decreased fALFF in children with GTCS was found in the main areas of DMN, such as the right temporal and the bilateral angular. GM volume of the right thalamus showed a negative correlation with the duration of epilepsy. Additionally, the fALFF value in the right hippocampus/thalamus showed a positive correlation with the duration of epilepsy. The consistent results using various analyses in the present study indicated that epilepsy may directly impair the brain structure and function in the thalamic-cortical/subcortical network of children with GTCS. The right thalamus plays an important role to reflect the chronic damaging effect of GTCS epilepsy. Children with GTCS showed special changes in the brain's structural and activity patterns, a measurable difference from previous results of adults with GTCS. Multivariate pattern analysis indicated that the abnormalities of GM volumes and fALFF values in the right thalamus showed some potential value to distinguish the children with GTCS from the healthy control group at the level of the individual. These results are likely to be valuable in explaining the clinical problems and understanding the brain abnormalities underlying this disorder.

## Author Contributions

YL, JW, and YW conceived and designed the experiments. YW and JW performed the experiments. YL and YW analyzed the data. YL and JW contributed reagents, materials, and analysis tools. WH was responsible for patient management and conceptualization of the study. YL and JW wrote and revised the paper.

### Conflict of interest statement

The authors declare that the research was conducted in the absence of any commercial or financial relationships that could be construed as a potential conflict of interest.
